# Visual detection of time-varying signals: Opposing biases and their timescales

**DOI:** 10.1371/journal.pone.0224256

**Published:** 2019-11-14

**Authors:** Urit Gordon, Shimon Marom, Naama Brenner

**Affiliations:** 1 Faculty of Medicine, Technion, Haifa, Israel; 2 Faculty of Chemical Engineering, Technion, Haifa, Israel; 3 Network Biology Research Lab, Lorry Lockey Interdisciplinary Center for Life Science and Engineering, Technion, Haifa, Israel; Johns Hopkins University, UNITED STATES

## Abstract

Human visual perception is a complex, dynamic and fluctuating process. In addition to the incoming visual stimulus, it is affected by many other factors including temporal context, both external and internal to the observer. In this study we investigate the dynamic properties of psychophysical responses to a continuous stream of visual near-threshold detection tasks. We manipulate the incoming signals to have temporal structures with various characteristic timescales. Responses of human observers to these signals are analyzed using tools that highlight their dynamical features as well. Our experiments show two opposing biases that shape perceptual decision making simultaneously: positive recency, biasing towards repeated response; and adaptation, entailing an increased probability of changed response. While both these effects have been reported in previous work, our results shed new light on the timescales involved in these effects, and on their interplay with varying inputs. We find that positive recency is a short-term bias, inversely correlated with response time, suggesting it can be compensated by afterthought. Adaptation, in contrast, reflects trends over longer times possibly including multiple previous trials. Our entire dataset, which includes different input signal temporal structures, is consistent with a simple model with the two biases characterized by a fixed parameter set. These results suggest that perceptual biases are inherent features which are not flexible to tune to input signals.

## Introduction

Human perception is a complex process which involves both sensory and cognitive components; it is thus sensitive to many factors other than the sensory input itself. As a result, responses to seemingly simple and controlled stimuli can be non-reproducible and fluctuate among repeated experiments. These fluctuations are inherent to Psychophysical performance; given the ubiquity of noise in the nervous system [1], they could be plainly interpreted as such. However, recent studies suggest that they represent the influence on perception of factors other than the input: context [2–4], history [5, 6], perceptual memory [7], attention [8] and expectation [9].

Temporal context and past history, both of the stimulus and of the response, influence perceptual decision making [10] with and without feedback on performance [9, 11, 12]. Two opposing effects have been documented: On the one hand, observers tend to repeat previous responses, or to estimate signals as being similar to those previously perceived [5, 13–17]. Such effects are predominant when stimuli are weak, near perceptual threshold [13–15]. On the other hand, a negative bias appears towards perceiving a signal as opposite or different from the previous ones. This effect is usually seen after exposure to a strong or sustained sensation, and can be manifested as an overshoot in the estimation of a new or different stimulus [18, 19].

In many psychophysical experiments, signals are presented in a random uncorrelated sequence [10, 16, 20–22]. Even this simple design has revealed trial-to-trial response correlations [21, 23–26], and was used to study the biases and temporal context effects themselves [10, 16, 20–22]. Most studies examined each trial relative to one or two previous inputs [9, 16, 27] or responses [13], or relative to a priming stimulus [17, 28]. Perception in a more natural setting entails continuously varying inputs that can be correlated in time. One may expect that history and context dependence of perception be coupled to these temporal structures, but the nature of this coupling is still not well understood. In particular an outstanding question is to what extent human biases and history dependence are flexible variables that can tune to the properties of the sensory environment. In the current study we address these questions by manipulating input signals to have various temporal structures, and by employing dynamic analysis methods that highlight time-dependent aspects of the data. While the elementary detection task is seemingly simple, forming continuous streams of such tasks with various correlations and addressing the time dependencies of both input and output, reveal nontrivial facets of perception. It moreover brings the Psychophysical experiment a step closer to natural conditions.

Our results show that in a near-threshold detection task, human observers exhibit simultaneously both positive and negative biases in the same experiment. This is in-line with previous experiments with different setups. Our experiment design uncovers the temporal nature of these two biases, showing they act on different timescales: in the short term, responses are biased to be similar to previous ones. In the long term, a bias towards spontaneously switching the response is found. These two opposing biases are found to coexist in every stream of detection tasks we performed.

Our experiments shed light on the nature of the well-known tendency of humans to repeat their previous choice: this tendency seems to be fixed and not modulated by the input signal. Moreover, it is stronger for rapid than for prolonged response times, suggesting its origin is not sensory but rather further up in the hierarchy, e.g. in decision or motor response. The adaptation bias depends on multiple previous inputs and thus is sensitive to longer timescales. However due to limited statistics its properties is more difficult to characterize. Our data also sheds light on the relation between the biases and the incoming signals: our entire data-set is faithfully reproduced by a simple model with a fixed set of bias parameters. This supports a picture where both biases are not modified over behavioral timescales to account for different input statistics.

## Materials and methods

### Experiment design

#### Elementary visual task

**Fig 1:**
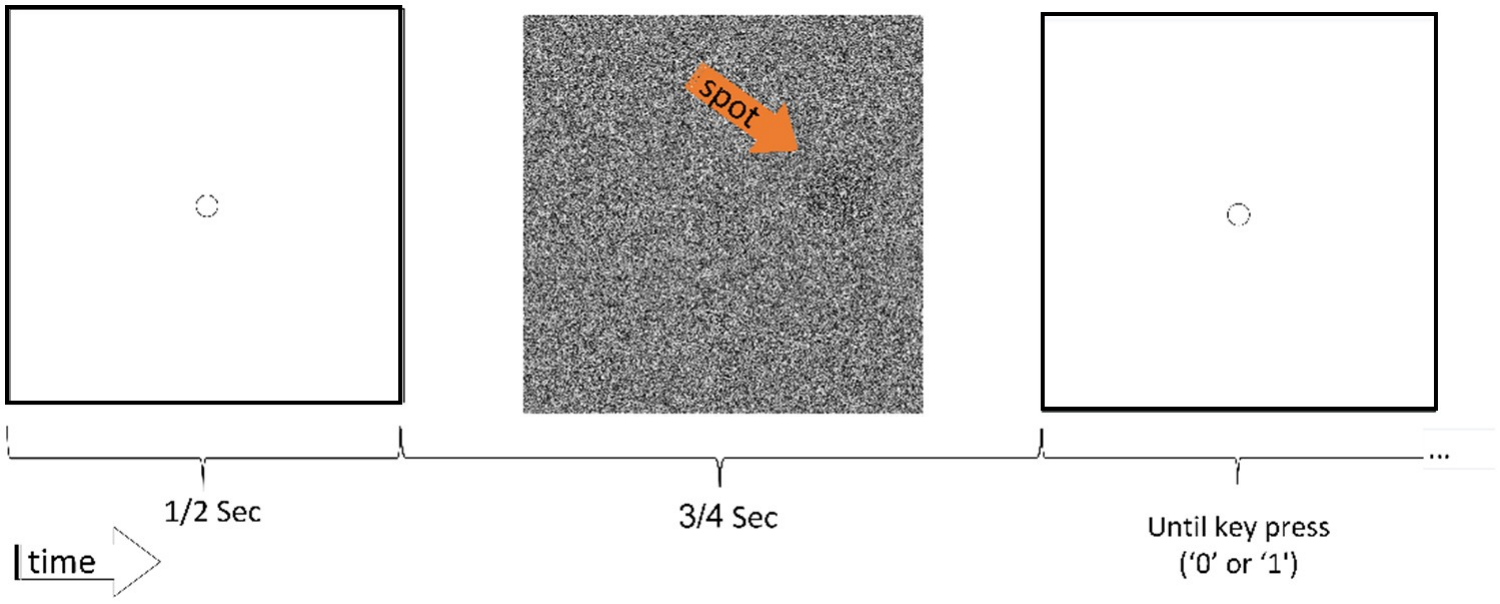
A stimulus trial starts with a blank screen with a fixation circle. After 500ms the stimulus appears for 750ms: a circular spot with random black and white pixels, darker than the random black and white background. Then, the blank screen returns and remains until the observer reports whether or not a spot was detected. The input level is the probability of a black pixel in the circular spot (here 0.6), whereas the background has probability 0.5. The spot, appearing at a random angle at a distance 150 pixels from the center, is marked here with an arrow for illustration.

The experiment contained a series of elementary visual detection tasks presented to human observers. The background screen showed a pattern of 500X500 black and white pixels each drawn independently with probability 0.5 to be black. The pixels occupied 135X135 mm of the screen, and observers sat approximately 50 cm from the screen. A new background with the same statistics was generated for each trial. On top of this background a darker circular spot, 60 pixels in diameter, was presented, 150 pixels from the center at a randomly chosen angle. The spot was composed of black and white pixels with probability larger than 0.5 for pixels to be black. This probability is referred to as the **input level**. In the example given in Fig 1 the input level was 0.6; this is well within the detectable range of most observers. This task design is similar to that used in [4] to study the effect of closed-loop feedback control on visual response fluctuations. Each trial began with a visual reset period of 500ms, in which a white screen with a fixation circle in the center was presented. The stimulus was then presented for 750ms, after which the white screen returned (Fig 1). The observer was instructed to report with a key-press whether or not a spot was detected (‘1’ or ‘0’ respectively). Pressing the response key immediately initialized the next trial. The experiment was self-paced since response time had no upper time limit. Responses were accepted also during stimulus presentation without shortening the trial.

This study was reviewed and approved by the Technion Review Board according to Technion regulations on ethical conduct in performing behavioral research on humans.

#### Temporal structure of stimulus

**Fig 2:**
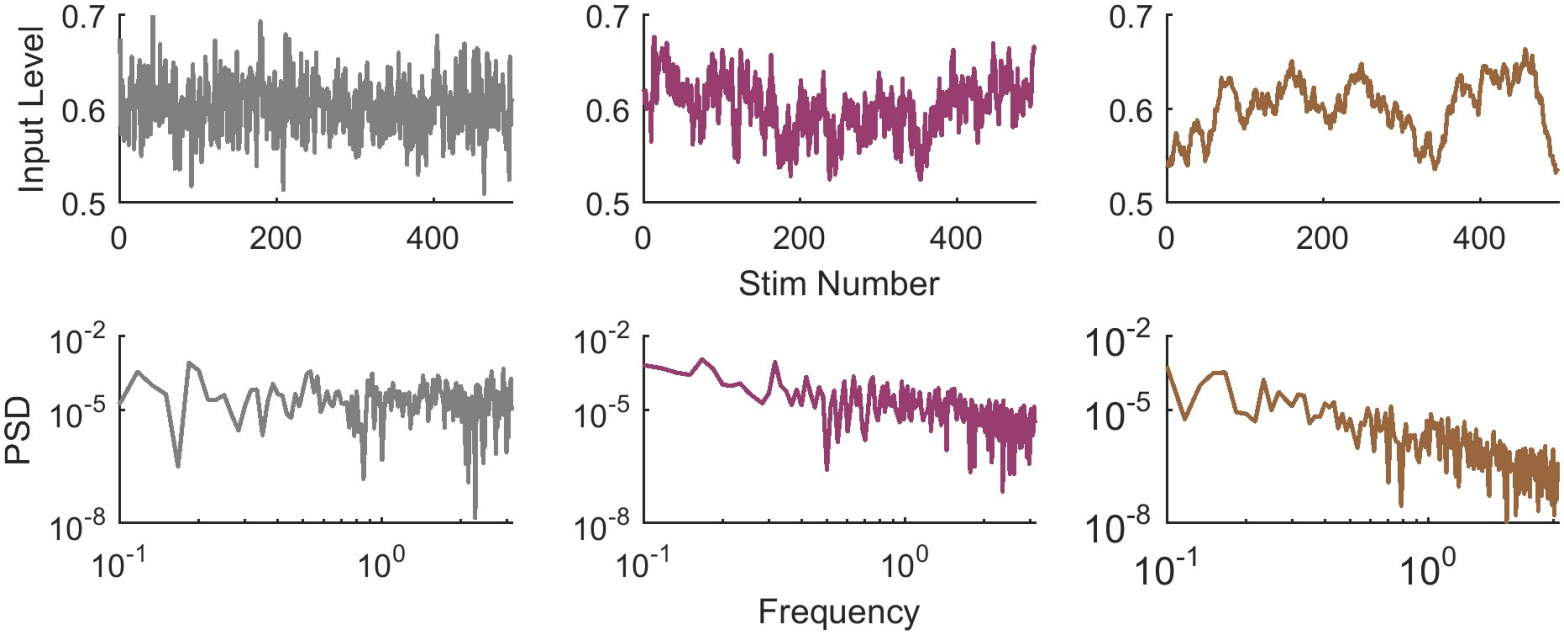
Temporal structure of stimuli. Examples of input levels in their temporal order (top panels), and their corresponding power spectral densities (PSD, bottom panels), for the three types of stimuli used in the experiments. In the “White” stimulus (left; grey lines), consecutive input levels are independent and the PSD is flat. In the “Pink” stimulus (center; pink lines), consecutive input levels are correlated and the PSD decreases with frequency. The “Brown” session (right; brown lines) varies extremely slowly, consecutive input levels are highly correlated and the PSD decreases sharply.

Input levels on consecutive trials were drawn from a normal distribution with fixed mean and variance, and with different temporal correlations: a “White” stimulus was created by drawing the level independently for each trial; the “Pink” stimulus contained correlations, namely the changes in input levels among consecutive trials varied slowly; and finally the “Brown” stimulus changed even more slowly in time. Examples of 500 input levels for each case are shown in Fig 2 (top panels). These three types of temporal structures can also be characterized by their Power Spectral Density (PSD), shown in the lower panels of Fig 2. The Pink stimulus has a PSD decreasing as $\frac{1}{f}$, while the Brown decreases as $\frac{1}{f^{2}}$.

#### Experimental protocol

**Fig 3:**
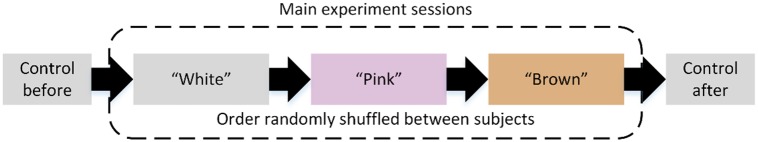
Experimental protocol.

The entire experiment consisted of 3 main sessions, containing 500 consecutive trials each, with the three temporal structures described above. The sessions were presented in random order. Two additional control sessions were conducted before and after the main sessions (Fig 3).

The first control session consisted of 100 trials, with stimulus levels independently drawn from a Gaussian distribution with mean 0.595 and standard deviation (STD) 5% of the mean. Responses were characterized by a psychometric curve (see next section for details). Analysis of this session was used to adjust the average input level in the following sessions to be at the individual threshold, i.e. the 50% detection level, and the standard deviation to be 5% of this value. This ensured a fair comparison among observers, with the stimulus levels being very close to the individual detection threshold throughout the experiment.

18 subjects aged 23-31, 9 females and 9 males, participated in the experiment. They had regular or corrected-to-regular vision and were not diagnosed as having attention deficit disorders. Two female subjects were excluded from the experiment for having extremely high positive responses (> 40%) for trials of very low input levels, which implied low credibility. The experiments were conducted in a dark room where observers sat alone in front of a computer screen. No feedback on the tasks was provided along the experiment. All participants signed consent forms, were paid for their time, and were naive to the purpose of the experiment.

### Statistical analysis—Extracting psychometric curve parameters

**Fig 4:**
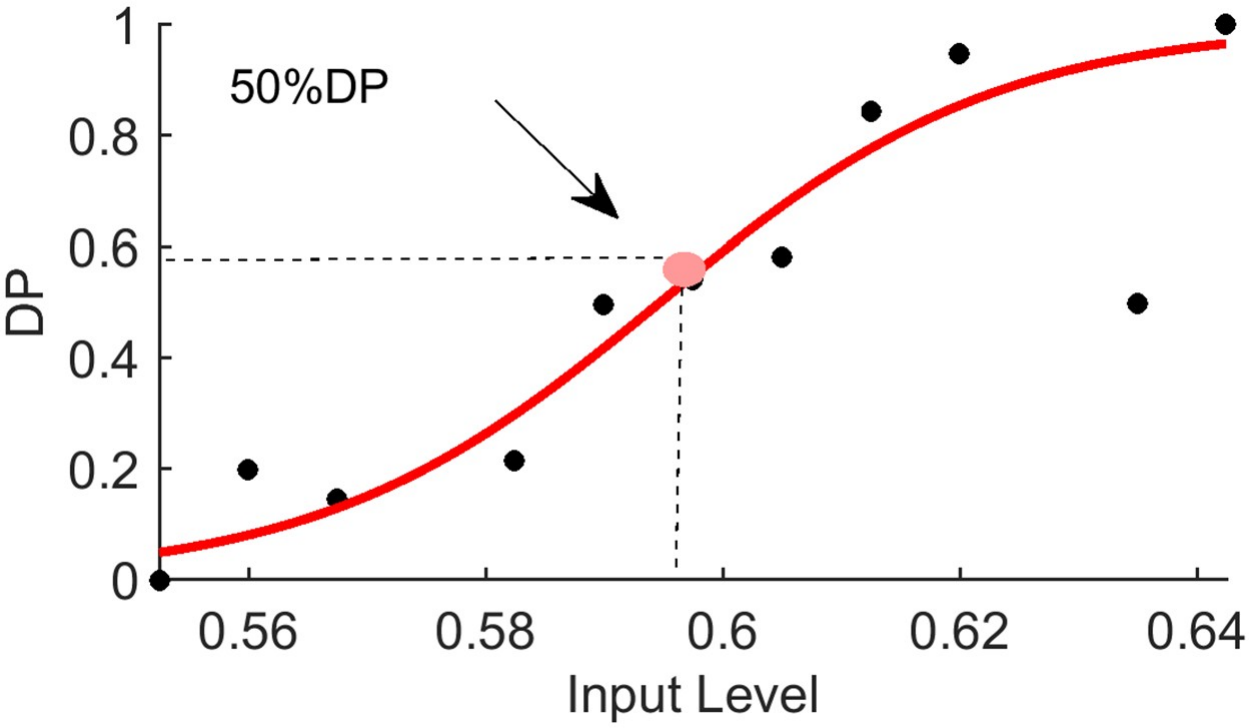
Psychometric curve parameters. The input level at which detection probability is 0.5 defines the threshold *θ*. The steepness of the curve at threshold is represented by the slope *k*, see Eq 1.

The Detection Probability (DP) was computed as the fraction of detected trials with input levels in bins of width 0.075 (Fig 4). A continuous curve of DP as a function of input level was estimated from these points using weighted curve fitting to a sigmoid function:

$$\begin{array}{c} DP=f\left(x;\theta ,k\right)=\frac{1}{1+10^{k(x-\theta )}} \end{array}$$ (1)

Where *θ* is the threshold and *k* the slope of the sigmoid. The weight of each bin in the fitting was determined by the number of samples in the bin. The fitting process was iterative using *Matlab* function for nonlinear fit (nlinfit.m).

### Model: Instantaneous observer and biases

**Fig 5:**
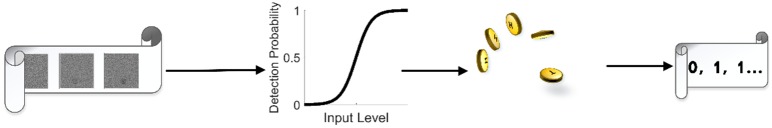
Instantaneous model observers were simulated to separate effects of the input structure from internal biases. Instantaneous model observers receive a stream of input signals (*x*_*i*_; left) which goes through a local sigmoid input-output relation *f*(*x*_*i*_) to define a probability of detection for each input (middle). The response is then determined by a coin-toss according to this probability resulting in a binary detection (right).

The use of correlated inputs in our experiments may have various effects on response statistics. It is critically important to disentangle these effects, which are properties of the input, from true biases of the observer. To this end, we make use of an instantaneous, bias-less model observer, and compare the measured results with simulations of such an observer exposed to the same type of stimuli used in the experiment. The instantaneous observer computes the DP by a fixed input-output function that acts on the current input: *DP*_*i*_ = *f*(*x*_*i*_), and then tosses a coin with this probability to determine whether the input was detected or not (Fig 5). The function *f* is a sigmoid as in Eq 1 with parameters *k* = 30, *θ* = 0.595, corresponding to the average estimated over all human observers.

Two opposing biases were tested by including them in the model and comparing their performance to the data. The Detection probability is then

$$\begin{array}{c} DP_{i}=f\left(x_{i}-A\right)+\delta P,\;\;A=\gamma \left({\bar{x}}_{i}-\theta \right), \end{array}$$ (2)

where the recency bias parameter magnitude is *δP* = 0.1 and its sign determined by the previous trial. The adaptation term *A* was modeled as a linear force balancing the recency bias according to past history of the input signal. Specifically, the past history trend ${\bar{x}}_{i}$ is defined as

$$\begin{array}{c} {\bar{x}}_{i}=x_{i}\left(1-e^{-\frac{1}{\tau }}\right)+{\bar{x}}_{i-1}\left(e^{-\frac{1}{\tau }}\right) \end{array}$$ (3)

with *τ* = 24 [time steps]. The force constant was chosen *γ* = 0.25. In principle probability was clipped at [0, 1] but with the stimuli and response functions used in our experiment this was not required. The effect of each bias separately was investigated, showing that both are needed to correctly reproduce the data (See ‘Models of Separate Biases’ and Figs D and E in S1 Text).

## Results

### Sharper psychometric curves for slowly varying inputs

**Fig 6:**
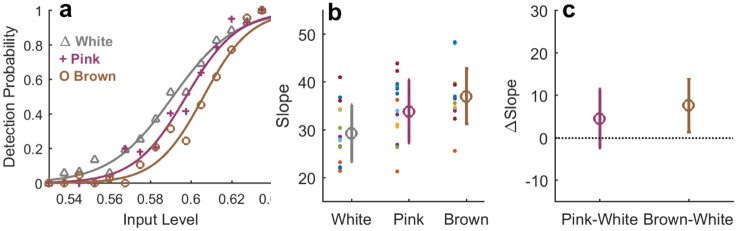
Slope of psychometric curve depends on input temporal structure. a) Example of psychometric curve for a single observer, *IR*_*F*_23. Symbols: binned detection probability, lines: fitted sigmoid. Color code marked in legend. b) Estimated slopes of psychometric curves for all observers (each colored dot is an individual). On average, the slope increases for more slowly varying input signals: 29.3 ± 2.5 32.8 ± 4.2 36.5 ± 2.7 for White, Pink and Brown respectively. Errorbars mark the standard deviation (STD) across observers. c) Individual slopes relative to White session: Slope estimated for White signal is subtracted from slopes of the other signals for each observer individually. Statistical T-test performed, significant changes were found with p-values 0.02 and 0.0003 respectively.

We first characterized the observers’ performance to the different temporal signals by estimating the psychometric curve for each of the stimulus types: White, Pink and Brown. The psychometric curve is based on the fraction of stimuli detected as a function of input level, and represents the response to the momentary input averaged over the entire experiment. Fig 6a shows an example of the three psychometric curves computed for one observer. Sigmoidal fits to the data are shown in solid lines, defining two parameters for comparison among observers: the slope *k* and the threshold *θ*. These were extracted for all experiments as described in Statistical Analysis section. The curve is most shallow for the White stimulus, where input levels are presented independently at each trial. It becomes sharper for the Pink stimulus with temporal correlations, and is sharpest for the Brown stimulus which varies most slowly. The slopes for all observers are shown in Fig 6, where the average is seen to increase with the signal correlation: on average over all observers,

$$\begin{array}{c} k_{White}<k_{Pink}<k_{Brown} \end{array}$$ (4)

Since variability between observers was high, we considered the relative change among stimulus types for each individual separately. For each one we subtracted the slope of the psychometric curve obtained for the White stimulus from those of the correlated stimuli, Pink and Brown. The result shows that the per-observer slope of the psychometric curve in response to the White stimulus is lower than that of Pink or the Brown (Fig 6c). The slope obtained for the Brown stimulus session was higher on average than of the Pink, but less significantly so.

The observer of Fig 6a exhibited also a shift in detection threshold *θ* between input regimes. However, this was not a consistent effect among all observers, neither on average nor when subtracting the thresholds individually. These data are presented in ‘Fixed threshold in all stimulus regimes’ and Fig B in S1 Text. Consistent with this observation, the total detection probability of individual observers also did not change systematically among the different stimulus regimes.

The dependence of psychometric curve slope on temporal stimulus properties highlights the interplay of those properties with perception. Since the different stimuli have the same overall distribution of input levels (see Methods), if perception were instantaneous the same psychometric curve would be measured for all of them. The different curves therefore reflect additional variables, related to the sequence of presentation, that affect perception. On a qualitative level, a simple statistical argument is sufficient to show that a fixed bias towards repeating the previous response results in an increased slope for correlated inputs (See SI). Our main goal in what follows is to further characterize these additional variables quantitatively—the biases, their timescales and origin, and their dependence on input. To this end we need to go beyond the psychometric curve and analyze responses in temporal context.

### Probability of alternating responses

Psychophysical experiments with sequences of independent random signals have revealed a “positive recency” effect: the current response tends to be similar to the previous one [5, 11, 22]. A quantity which measures the magnitude of this effect in binary responses is the probability of alternation (POA), defined as the fraction of reversals in a binary string:

$$\begin{array}{c} POA\equiv \frac{number\;of\;alternations}{number\;of\;trials-1} \end{array}$$ (5)

Where *‘number of alternations’* means the number of changes, between any one type of response and the other. For a random, symmetric uncorrelated binary string, POA is expected to be 0.5; lower values correspond to strings with longer streaks.

The number of alternations is affected also by the input: if it is correlated in time and varies slowly, it has long stretches of below- or above-threshold regimes, that will result in long streaks of ‘0’ or ‘1’s, respectively. Therefore the POA will be lower for the more slowly varying inputs even for an instantaneous observer with no biases, reflecting a property of the input itself. To disentangle this input-dependent effect from possible bias and history-dependence of the observer, we measured the POA of human responses in comparison to those of instantaneous model observers exposed to the same inputs (see Methods).

**Fig 7:**
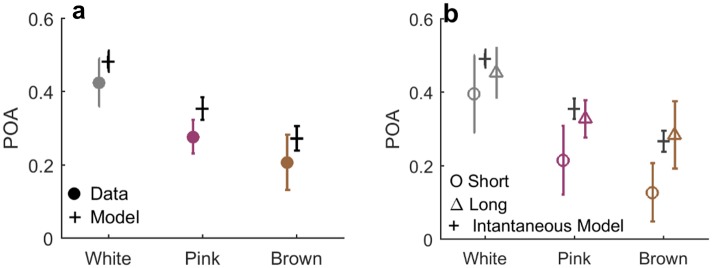
Repetitive bias quantified by Probability of Alternations (POA). a) POA computed for human observers (filled circles) and for 15 instantaneous model observers (Black +’s). Means are [0.43,0.28,0.21] for the model and [0.49,0.35,0.27] for the data, for White, Pink and Brown inputs respectively. Errorbars: Standard Deviation. Two sided T-test was performed between the groups of values showed strong significant difference (p-values: 0.0007,<0.0001,0.01). b) POA strongly depends on response time in all stimulus regimes. Trials were separated to shorter and longer than median for each observer, and POA computed for each group separately. The slow trials (marked Δ’s) exhibit a POA similar to the bias-less observer (marked black +’s), while the fast trials (marked O’s) show a much stronger repetitive bias and are largely responsible for the average effect seen in a.

Fig 7a shows the POA computed for a group of instantaneous model observers, simulated with inputs from each of the three stimulus regimes, marked in black. As expected, the white stimulus induces a chance-level POA of approximately 0.5, while the slower stimuli elicit less alternation even without any history-dependence on the observer’s part. The same figure shows also the POA computed from our experimental data of human observers, marked in colored circles, showing values lower than the instantaneous model for all stimulus regimes. The decrease in POA relative to the instantaneous model observer quantifies the inherent tendency of the human observers to repeat the same response as the previous one, generating streaks longer than justified by the input. The effect is similar in magnitude across all three stimulus regimes, suggesting that this bias is fixed and not modulated by the correlation time of the input signal.

What is the origin of the recency bias—is it sensory, related to decision, or rather to motor response? To shed light on this important question, we investigated the relation between response time and POA. Fig 7a shows the parsing of all data shown in Fig 7b according to response time, forming two equal-sized groups of trials with long and short response times (defined for each observer separately). The figure reveals that trials with faster response exhibit a significantly stronger repetitive bias. This implies that additional processing time before responding resulted in a ‘correction’, or compensation, of the bias towards the performance of the instantaneous simulated observer (black crosses). This in turn suggests that repetitive bias can be ‘undone’ and therefore probably does not originate from the sensory part of processing, but from a later stage.

### Dependence of response on previous input

**Fig 8:**
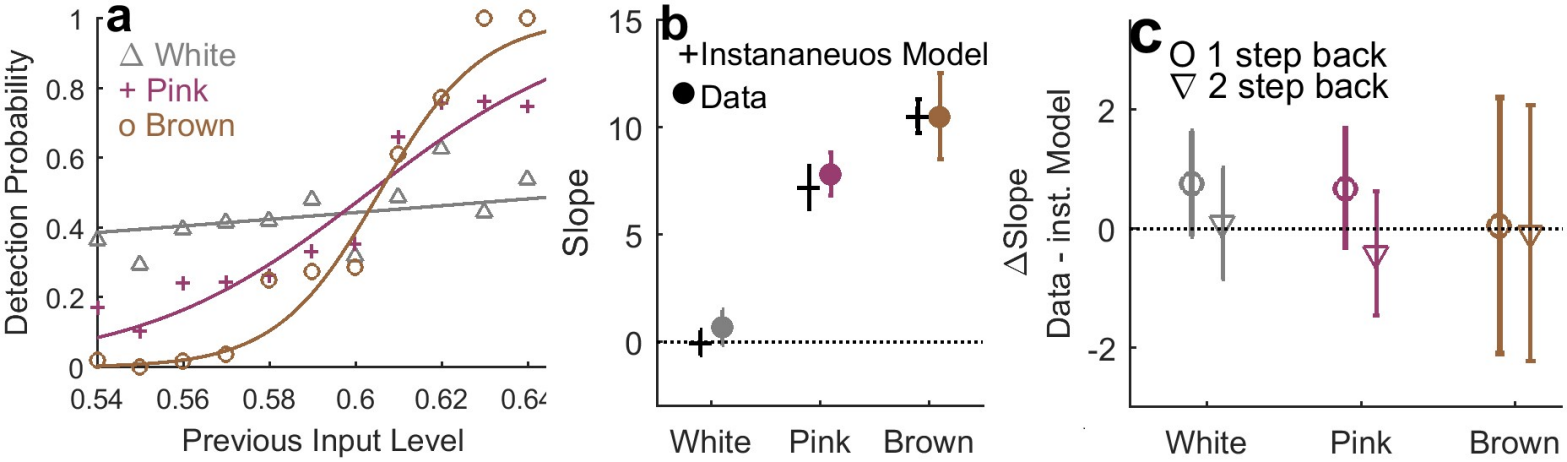
Dependence on previous input. a) An example b) Psychometric curves are formed by estimating the detection probability as a function of the previous input from experimental data. The curve becomes sharper for colored inputs partly due to the correlation between current and previous input. c) Comparison to unbiased instantaneous model observers exposed to the same inputs, reveals the effect of recency bias inherent to the observers, in white and pink inputs. In brown there is much larger variability between individual data values.

An additional angle on the recency bias may be obtained by examining directly the dependence between current response and previous input level. Fig 8a shows an example from a single observer, where the psychometric curve was computed with respect to the previous input for the three stimulus regimes. For a White input, any dependence on previous input level represents a bias of the observer; in contrast for the colored inputs, the appearance of a high previous input level is correlated with that of a current input level. Therefore once again, to disentangle input and observer effects, we compare the result of this analysis to those of an instantaneous observer. Fig 8b displays this comparison, in terms of the slopes averaged over all observers—human (colored circles) and model (black). The experimental data exhibit consistently a higher slope for the psychometric function, indicating a dependence on the previous input beyond input correlations. The difference between data and model (Fig 8c, circles) seem to be approximately constant in the White and Pink stimulus regimes, not showing a significant trend that follows the increase in input correlation. Note that the results for the Brown input suffer from higher noise level, since the input is so strongly correlated that these correlations dominate the dependence on previous input. These data are consistent with the notion that recency bias is not systematically modified by input correlations, in agreement with the POA analysis presented above.

The dependence on previous inputs may be further analyzed to reveal the timescale typical of the recency bias. We repeated the analysis of Fig 8a and 8b as a function of the one-before-last input signal. Fig 8c (triangles) shows that the difference in slope disappears and becomes insignificant. In particular the result for the White input clearly demonstrates that while the last stimulus has a measurable effect on the slope, the one-before-last does not.

**Fig 9:**
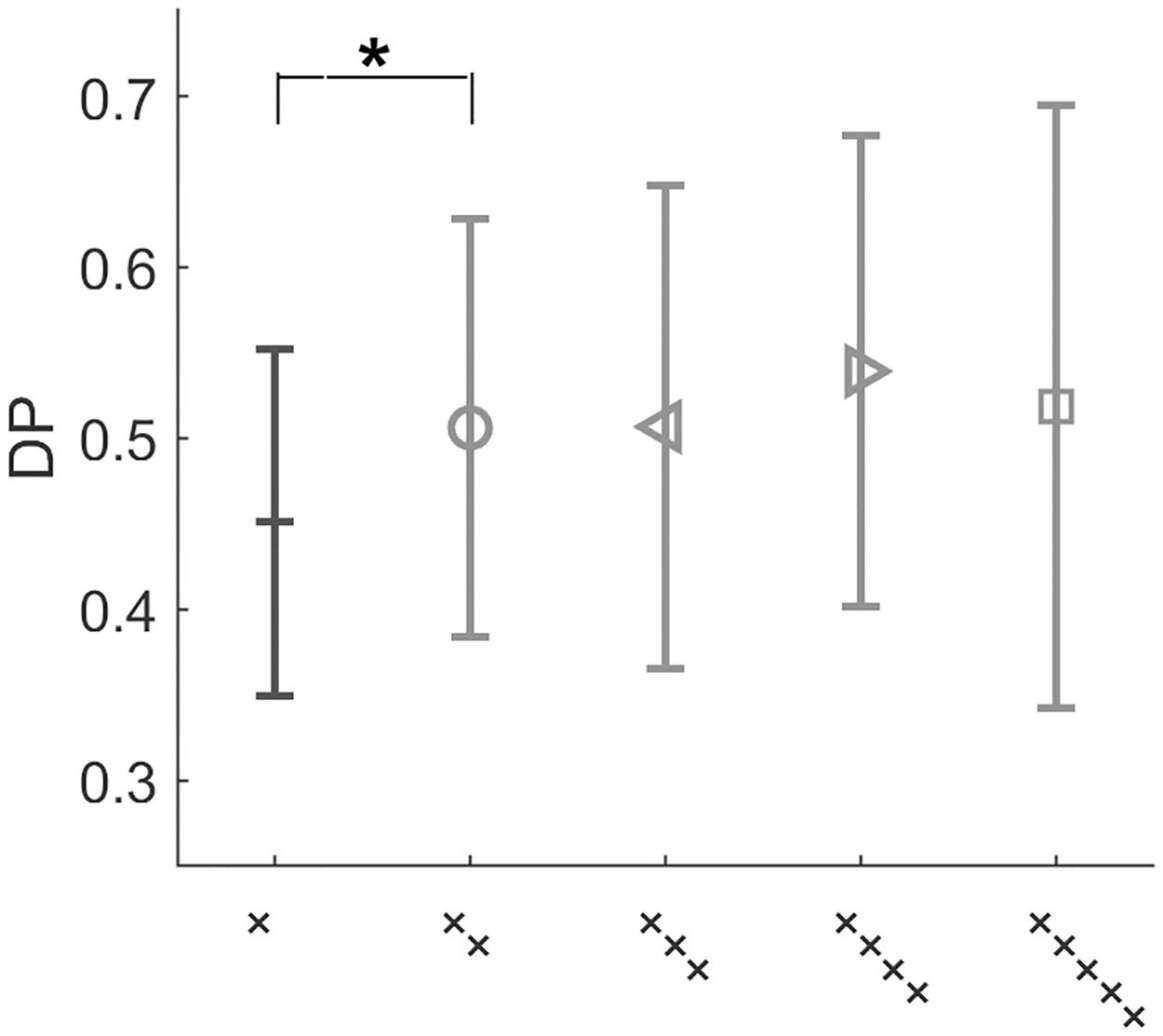
Dependence of response on previous response. Detection probability of current signal conditioned on previous positive detections for White stimulus. +”: DP of current input. ‘++’: DP conditioned on detection of previous input, etc. DP increases significantly (p-value: 0.009) when the last input was detected, but does not continue to increase significantly for detection events further into the past.

A complementary view of this effect can be obtained by considering the dependence on the current response on previous responses, without reference to the input signal. Focusing on the White input, we compute the detection probability of an input averaged over the entire experiment compared to an average conditioned on past responses. Fig 9 that only the immediately previous trial has a significant effect on the current detection, whereas events further back into the past do not have a consistent effect. This result, taken together with the dependence on past inputs, suggest that the sensitivity of positive recency to previous trials is limited in its temporal extent to approximately one trial back.

### Psychometric curves conditioned on input trends

We have seen that the detection probability depends on the previous trial, with an approximately constant effect for all stimulus regimes. The analysis presented above focuses on the previous trial itself, either its input or its outcome. However, within a temporal framework, it is plausible that perception will be sensitive not only to values but also to trends—changes over time.

Therefore we analyzed psychometric curved conditioned on properties of the input signal other than its instantaneous (current or past) values. Trials were divided into two groups depending on stimulus trend preceding the current one—decreasing or increasing trend.

**Fig 10:**
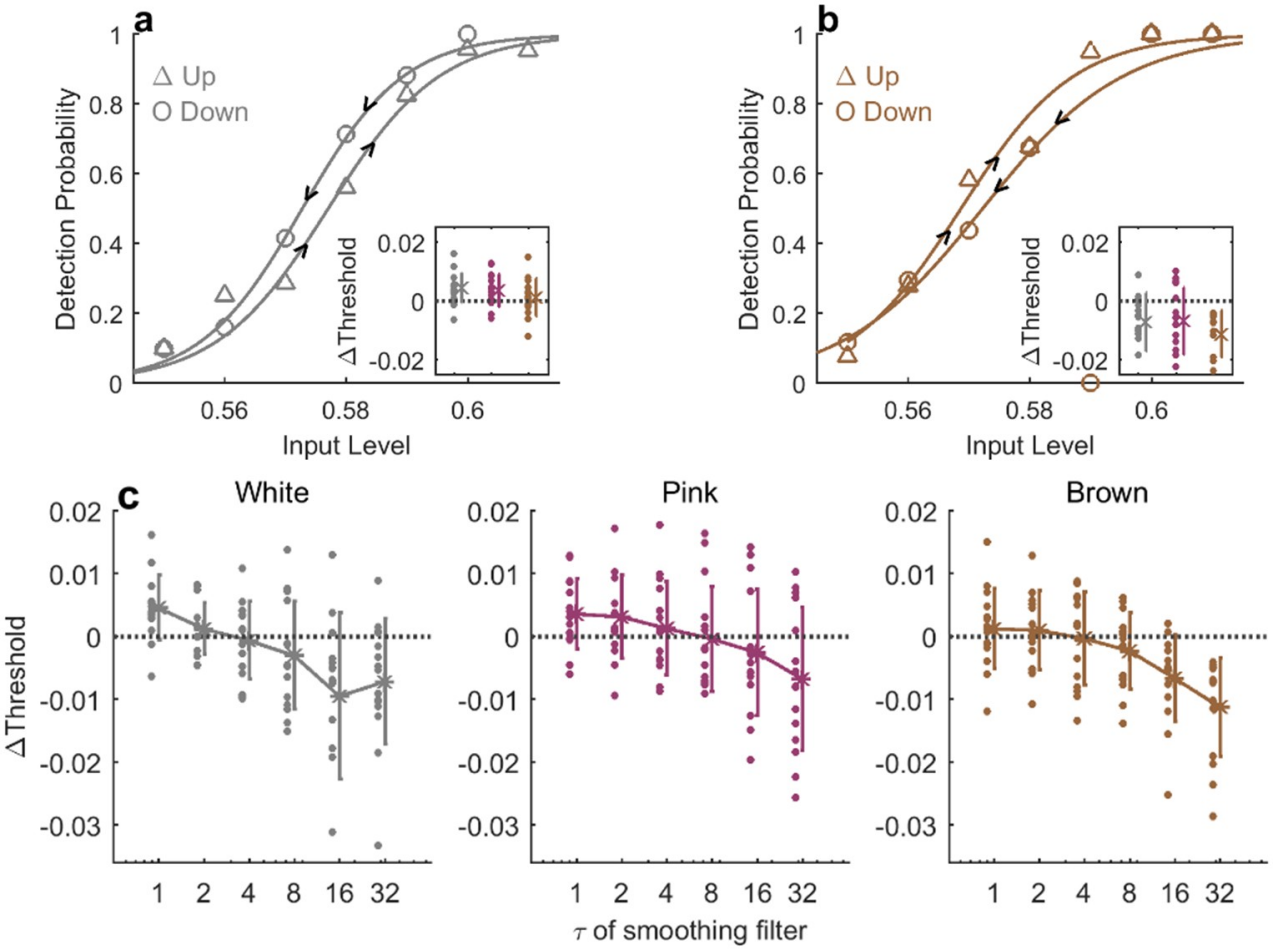
Positive and negative hysteresis in conditioned psychometric curves. a) Example of Up (Δ′*s*) and Down (O’s) psychometric curves, conditioned on whether the current stimulus is higher or lower than the previous one, in a White stimulus experiment for one observer. Data points: markers, sigmoid fits: lines. Inset: value of hysteresis, defined as the difference between thresholds, for all observers in the three stimulus regimes (White, Pink, Brown from left to right). b) The same analysis as in A, where Up and Down are determined by comparing the current input level to its trend over *τ* = 32 previous trials. Hysteresis is negative here: Up has lower threshold than Down. Example data is taken from Brown stimulus of the same observer. Inset: Hysteresis value for all observers in the three stimulus regimes with *τ* = 32. c) Hysteresis value of all observers in the three stimulus regimes, as a function of the timescale defining the past trend (*τ*).

Fig 10a shows the results for the White input signal, where the two groups correspond to the current stimulus being either of higher or lower contrast level than the previous one. Triangles for “Up” data, show trials in which the current stimulus level was higher than the previous one. Circles for “Down” data are constructed from trials in which the current stimulus was lower. Solid curves show sigmoidal fits to the two data sets. This analysis reveals a positive hysteresis: the Up curve has a higher threshold than the Down curve. Such positive hysteresis indirectly reflects a tendency to perceive the input as similar to the previous one: for the same input level, coming from high stimulus our detection probability is larger than coming from a previously lower one. It can be the result of a filtering operation, for example. The difference between the thresholds of the two conditional curves is shown in the inset, for all three stimulus regimes. It is seen that the effect is strongest for White input and decreases to an insignificant value for the Brown input. This can be partially explained by the fact that, in a slowly-varying stimulus, changes between consecutive input levels tends to be very small, whereas in the white stimulus they can be of any magnitude.

This analysis reveals a sensitivity to the input trend, but takes into account only the immediately recent trend with respect to the previous input. We generalize this analysis with longer timescale by comparing the current stimulus to the past history averaged over a time *τ*. This analysis may reveal the effect of the current input relative to expectation formed in a past time window. Trials are then divided into two groups depending on whether the current input value is higher or lower relative to the general past trend. Specifically, the input levels are filtered using an exponential filter of time constant *τ*, with time measured in number of trials; the result is then compared to the current input level.

Psychometric curves were estimated independently for the two groups, Up and Down, as before. An example is shown in Fig 10b, where a timescale of *τ* = 32 was used for defining the past input trend in a Brown stimulus experiment. In contrast to the previous plot, here we find a negative hysteresis effect, namely the threshold for Up trials (triangles) is lower than for Down trials (circles). A similar effect was found for all stimulus regimes. Negative hysteresis reflects an increased sensitivity moving from a weak to a stronger stimulus, which is usually referred to as adaptation. Such adaptation is typical to slowly changing environments that allow reliable prediction, with deviations from that prediction resulting in an enlarged reaction.

Quantifying the degree of hysteresis as the difference between thresholds of Up and Down curves, allows us to plot this difference for a range of *τ* values, corresponding to the length of history defining the trend. Fig 10c shows the result of this analysis, depicting all individual observers as dots, with averages and standard deviations marked. The trends are clear and similar for all stimulus regimes: in the short term a positive hysteresis appears, which decreases with *τ* until it eventually crosses over to a negative hysteresis for long times. The White stimulus reveals the largest magnitude of positive hysteresis, whereas for the Brown stimulus the negative hysteresis dominates. The crossover from positive to negative occurs at around *τ* = 4, without showing a significant systematic change of this value among stimulus regime. On a qualitative level we may conclude from these results that both processes, positive and negative biases, exist in human observers simultaneously in the same perceptual decision task. It appears that they emerge with different characteristic timescale—positive bias over short times and negative bias over longer times. The ultimate response pattern results from an interplay of the two and the nature of the stimulus.

### A model for biased perception

The experiments presented above suggest that, in addition to the input signal, two inherent opposing forces act to shape perceptual decision making on different timescales. On one hand, human observers tend to stick to their previous responses even when stimuli change. On the other hand, over longer timescales, an adaptation effect occurs which keeps the observer sensitive to changes. Using the model of Fig 5 as a basis, one may describe these two effects as modulations of the instantaneous input-output function.

We model the positive recency by adding a small probability bias *δP* to the output, positive if the previous input was detected and negative if it was not. From our experiments it follows that positive recency can be “undone” with a longer response time, suggesting that this effect is late in the process—decision or motor—rather than sensory. This is also consistent with recent work showing that repetitive bias is not a sensory effect [29, 30]. Adaptation, on the other hand, can be described by a modification of the sigmoid threshold, or equivalently, by adding a bias to the perceived signal [31]. This adaptation effectively adjusts the threshold as a result of transiently high or low inputs.

**Fig 11:**
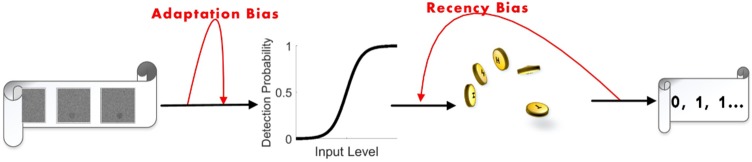
Model of perception with two biases. The backbone structure of the model (black arrows) is composed of a fixed input-output relation (*sensory process*) determining the probability of detection, and a coin flip *decision* based on this probability. Two biases modulate this backbone (red arrows): an *Adaptation Bias* varies the threshold based on the input history; and a *Recency Bias* modifies the final response based on previous one.

The general structure of the model is depicted by red arrows added to the instantaneous model backbone in black (Fig 11). Together these two biases give the detection probability *P*_*i*_ of Eq (2). Using this model we generated a set of 15 observers, and simulated their responses with the same protocol as the experiment. Input signals were synthesized in the three stimulus regimes, presented to the model observers, their responses (0/1) recorded, and the sequences of input and output were analyzed similar to the experiments. We note that in all model simulations the observers had the same parameters, and therefore variability between them is smaller than between human observers.

**Fig 12:**
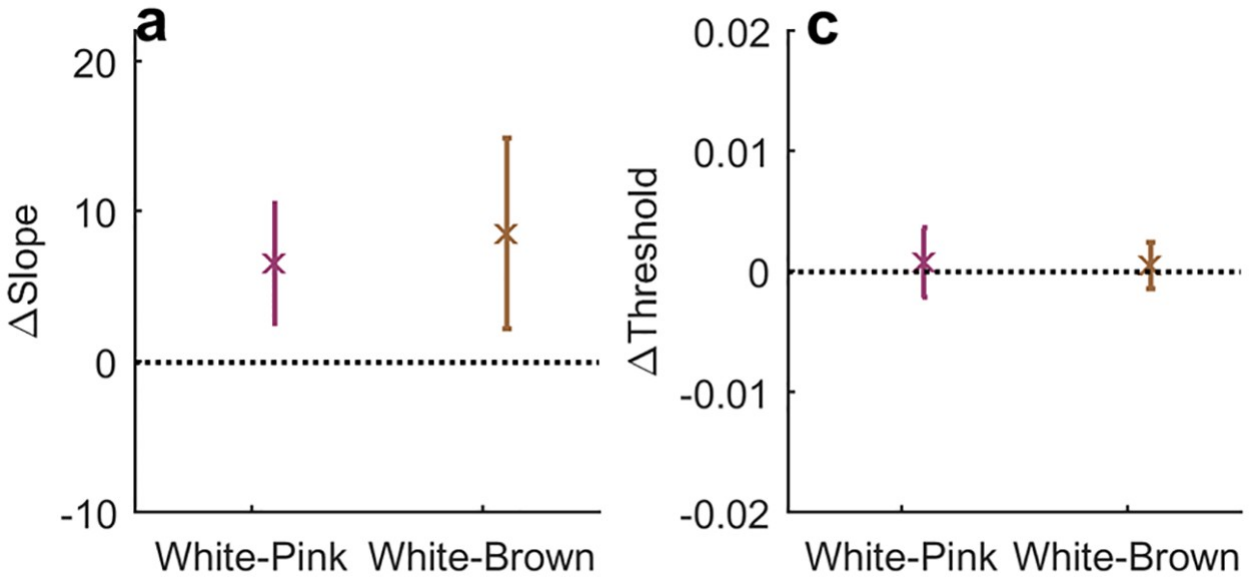
Empirical psychometric curve parameters for model observers. Model observers were presented with the three stimulus types, and their responses analysed to estimate empirical psychometric curves. Relative slopes a) and thresholds b) were computed by subtracting the corresponding parameters of the White stimulus from those of the Pink and Brown. Compare to experimental results in Fig 6.

**Fig 13:**
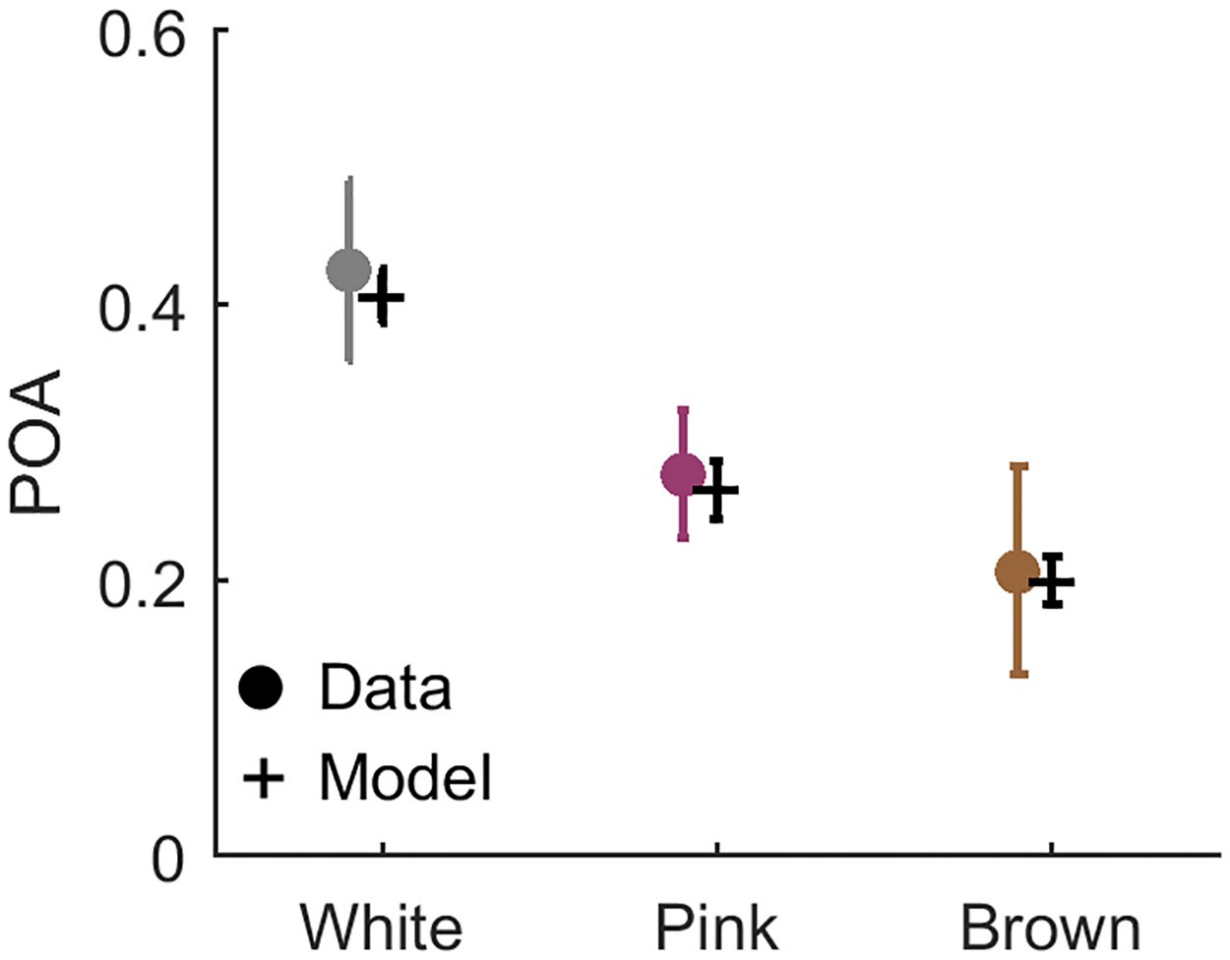
POA of model and data. Probability of alternation (POA) in the response of humans observers (filled circles) and of model observers (Black).

**Fig 14:**
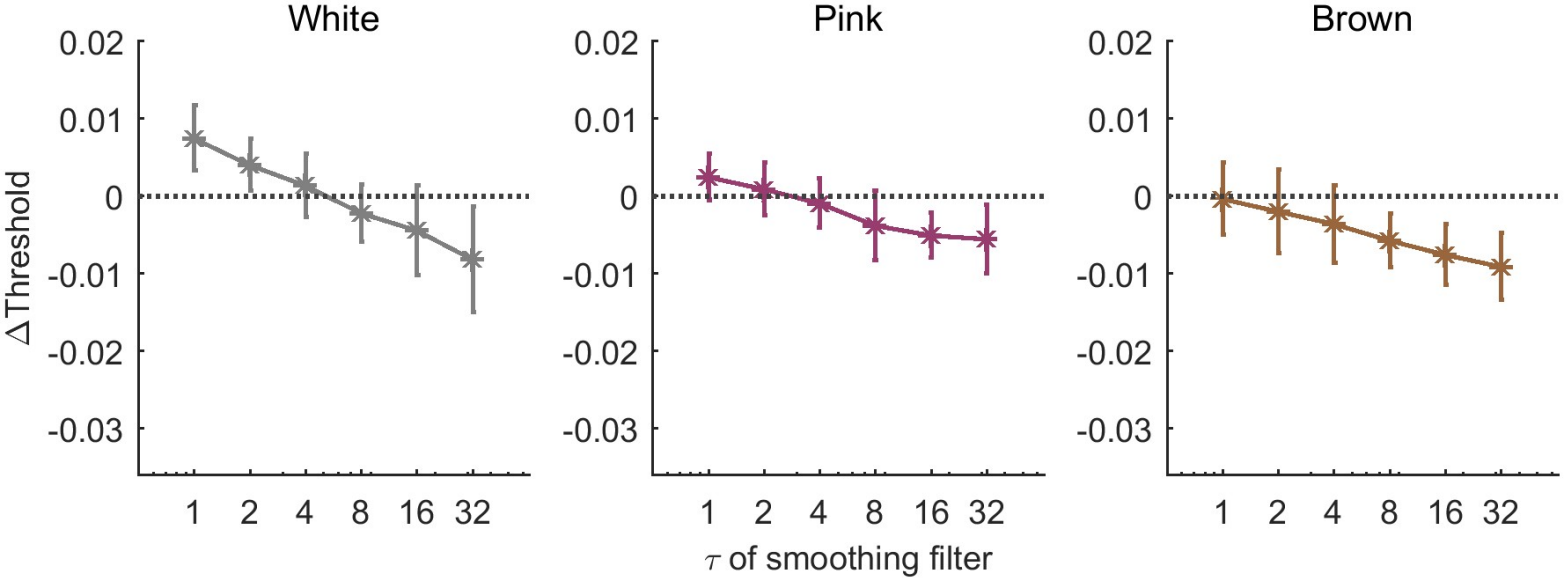
Hysteresis in model psychometric curves conditioned on stimulus trend. Hysteresis in model shows qualitatively the same behavior as a function of *τ*, the timescale used to define the stimulus trend as Up or Down, as human observers, for all stimulus regimes.

We first ask whether the model combining the two biases can generate the characteristics of the measured data. In particular it is important to understand whether all stimulus regimes can be described by the model with one set of parameters, or alternatively these parameters should be tuned to the input properties. This should give an indication whether biases of human observers are fixed or modified by inputs. The results, showing that the entire data-set could be reproduced with one set of model parameters, are presented in Figs 12–14.

First we used the responses of the model observers to construct their empirical psychometric curves. Although the input-output sigmoid function defined in the model (middle box in Fig 11) was fixed, the existence of history-dependent biases in the model together with different temporal structure of the inputs resulted in empirical psychometric curves that depended on the stimulus regime. Fig 12 depicts the parameters of these empirical curves for the two correlated inputs—Pink and Brown—after subtracting those computed for the White stimulus. The model produced sharper psychometric curves (larger slopes) for the slowly varying stimuli (Fig 12a), while the threshold values remain unchanged (Fig 12b). The effects are similar in direction and in magnitude to those found for human observers.

Next we considered the probability of response alternation (POA) averaged over the entire experiment, for the different stimulus regimes. Fig 13 shows the results for all model observers (black) together with the same quantities computed for the experiments on human observers (colored circles). They are practically indistinguishable, showing that the model captures correctly the tendency for positive recency equally well in all stimulus regimes.

The model also captured the empirical psychometric curves conditioned on the stimulus trends.

Fig 14 shows the hysteresis—difference in thresholds between the two groups—conditioned on the direction of stimulus change, defined over various timescales. This can be compared to Fig 10: the model shows the same general profile of hysteresis values as a function of the timescale used to define the trend in the stimulus. In particular a crossover from positive to negative hysteresis is found at longer integration times, which becomes dominant as the input correlation time increases from White to Brown.

Recent work has shown that biases can potentially confound with signal correlations to produce other apparent biases [32]. Our model enables us to test this possibility by simulating each one of the biases separately and comparing to the experiments. S1 Text, ‘Models of separate biases’, shows that a model with only positive recency bias (Fig D in S1 Text) has a strongly and linearly increasing threshold of the psychometric function, in contrast to the data which show a saturating effect. Furthermore, conditioning the data on trends in the past input shows only a small effect of positive hysteresis, without the typical crossover from positive to negative exhibited by the data. We therefore conclude that an observer with only positive recency, with the correlated inputs presented in our experiments, does not show results consistent with the human observers tested.

A model with only adaptation bias is similarly insufficient to describe our experimental results. For example the slopes of psychometric curves show the opposite trend than the data—they decrease as input correlation time become longer. We also show in S1 Text the results of a model with only adaptation bias (Fig E in S1 Text).

We conclude therefore that the minimal model describing our dataset as a whole must contain both positive recency and adaptation effects. In particular this last result of hysteresis that changes sign as a function of history timescale, requires both biases. This establishes that both are indeed properties of the observers and not an artifact of the signal correlations.

Other models of the history-dependent effects that differ in details were also tested. The adaptation bias could be made to depend on the output rather than the input. The results of model observers following these processes were similar to the one presented above. Since our data cannot distinguish the source of the adaptation bias, both of these models should be considered equally consistent. Taking into account recent fMRI experiments indicating these biases are formed in different brain regions [33] suggests favoring the first one.

Another possible combinations was also tested, in which both biases modulate the decision processes (S1 Text); however, this was found to provide a slightly worse fit to the data, and moreover was less robust, i.e. more sensitive to the choice of parameters, and it was more difficult to find a set of parameters that fit the entire data-set. These alternative models are sketched in S1 Text, ‘Other models tested’ and Figs F and G in S1 Text.

## Discussion

In this study we have used temporally structured stimuli to investigate dynamic aspects of biases in visual detection. The task involved sequences of near-threshold visual stimuli; observers were asked to report detection / no detection, and no feedback was provided. While this elementary task is simple in itself, the sequence of input levels (contrast level for detection of a circle on a background) varied in time in a nontrivial way, spanning three stimulus regimes: White—where consecutive trials were independent; Pink—where they were positively correlated in time; and Brown—the most slowly varying succession of inputs.

Characterizing the detection experiments first by a static psychometric curve, we found that responses to more slowly-varying stimuli exhibit a sharper curve (Fig 6), although in all cases the overall distribution of stimuli was the same. This result clearly demonstrates that a static response curve does not tell the whole story of how perception is formed. Indeed, previous work had already found that perception depends on sequences of events in various contexts and modalities [5, 11, 22]. A bias towards repeating a previous response is sufficient to induce a dependence of the psychometric curve slope on input structure.

To shed light on properties of the biases, we analyzed the data using methods that emphasize history dependence. For example, dividing the experiments to groups conditioned on some sequence of events, and computing average response statistics or full psychometric curves for each of them separately. Using this approach, we could identify two opposing biases affecting perceptual decision making simultaneously on two separate timescales. First, a positive recency effect: human observers tend to repeat their previous judgment of the stimulus beyond chance, regardless of the input itself [17, 23]. This bias was manifested across all stimulus regimes by a decreased probability of alternating response over the entire experiment, when compared to a static observer (Fig 7). On a trial-by-trial basis it was reflected as a dependence of the detection probability on the previous input value, beyond correlations dictated by the input stream (Fig 7a). Comparing to trials two (or more) steps back, we found no significant dependence neither on the input (Fig 8) nor on the response (Fig 9). Analysis of the positive recency relative to an instantaneous, bias-less model observer revealed that the magnitude of the effect does not vary systematically across stimulus regimes. While the existence of a positive recency effect has been known for many years, it remains elusive in terms of its mechanisms and functionality; our experiments shed new light on these aspects. First, analysis of response as a function of previous stimuli and of previous responses, suggests a short—approximately 1 trial—timescale for the recency bias, indicating that response is mainly affected by the previous trial.

Second, using the recorded response times we found that the recency effect is primarily present in trials where the observers respond rapidly, and almost disappears when response times were long. The fact that this bias can be ‘undone’, suggests that it does not originate in the sensory part of the perception-action hierarchy, but rather in the later stages, either decision or motor [30, 34]. The fast timescale associated with this bias suggests the involvement of some automatic part of the response, such as the motor part [34].

Third, we found in several analyses that the magnitude of the positive recency bias does not change across different input regimes.

A second and opposite tendency, to change the response, was also revealed in the same set of experiments.

This effect was manifested in the response curves conditioned on the input trends over long times, resulting in negative hysteresis (Fig 10b).

Since adaptation after-effects can be sensitive to spatial location of stimuli, we tested whether overlapping spots were the source of this effect in our experiments. This is not the case (See S1 Text, ‘Spatial Effects’, and Fig H in S1 Text). We note that negative after-effects are found primarily in the regime of strong input stimulus, whereas the current experiments were performed near detection threshold.

The adaptation bias can be understood as follows: if the current input was low relative to the recent long-term average, in a slowly varying input, this would imply that the input has been high for a while. Therefore, the shift of the conditional curve to higher values implies an adaptation of the threshold to the recent statistics, moving it to the center of the incoming signal. In signal processing this can be rationalized as an optimal utilization of limited dynamic range of response to best code the incoming signal [35, 36]. In higher organisms long timescales were suggested to be a reflection of the system complexity [37, 38], with several possible mechanisms. Our data is insufficient to distinguish between possible underlying mechanisms; this important point remains open for future research.

Both positive and negative biases have been reported in previous psychophysical experiments. However only recently, Fritsche and el. [39] have shown the existence of two opposing biases simultaneously in a near threshold visual task. Our results corroborate this finding and further establish it; since experimental settings vary, this is a significant result. However, several details related to the quantitative features of the two biases still await experimental clarification, as they seem to differ between recent reports (see [29, 30, 39]). We assume that these differences depend on the setting and the nature of the task.

A question of interest is whether the timescale of both biases are flexible and locally tuned to varying environmental features. To answer this question, we constructed a simple model that captures how the two biases affect visual perception as an entire process, starting from sensory input to decision and action. All experimental results were reproduced using a fixed set of parameters, with a gap in timescales between the two biases. This suggests that the biases are fixed, not tunable to environment. Choosing the model parameters did not require very fine tuning; this in turn suggests that fitting the model to the data does not result in a pronounced maximum at some special values.

In a more general context our study offers a methodology which my be useful in future studies. In terms of experiment design, the input signal is regarded as a continuous stream and utilizes structured stimuli to create history-dependence. This approach allows dependence on history to surface on several timescales that were not inserted “by hand” into the experiment. On the complementary side of data analysis, we have used conditioning on various sequences of events with different characteristic timescales to expose the relations between temporal input and temporal output. These methods can be easily implemented in other sensory modalities and tasks.

## Supporting information

S1 Text Additional analysis of the experiments and alternative model versions with their comparison to the data. (PDF)
