# Supplementary material for: Visual detection of time-varying signals: Opposing biases and their timescales
Source: PLoS One. 2019 Nov 14;14(11):e0224256. doi: 10.1371/journal.pone.0224256 (PMC6855422; doi:10.1371/journal.pone.0224256)
Supplement: S1 Text — (PDF) [file pone.0224256.s001.pdf]

Paradigm validation - the task involves no learning

Control sessions before and after the main experimental sessions were used to verify that no learning was involved. Three parameters were tested: the two parameters of the psychometric curve, the threshold θ and the width k, and the total detection fraction over the session. As seen in Figure A, there is no significant change in any of these parameters before and after the experiment.

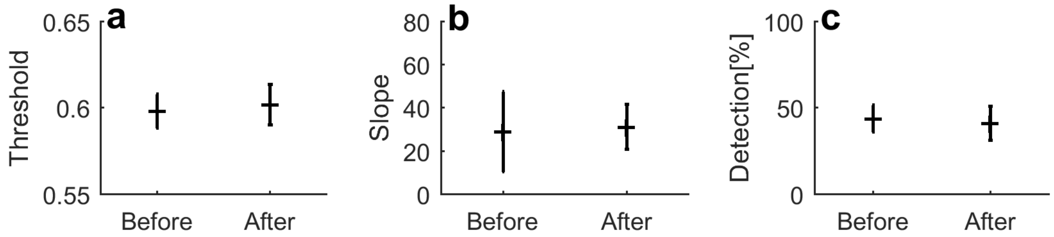

Figure A: Performance comparison before & after experiment. the parameters that were compared are (a) Threshold, (b) Slope and (c) Total detection rate. Errorbars marking mean and standard deviation across observers. No significant change was detected in any of the parameters.

Fixed threshols in all stimulus regimes

Comparing thresholds of psychometric curves fit to individual observers, we see no change in threshold across the three stimulus regimes. This is true also on average over all observers.

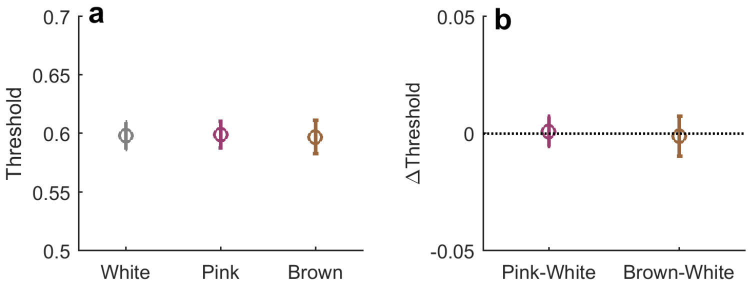

Figure B: No change in threshold between differently correlated input signals. (a) Thresholds of psychometric curves for White, Pink and Brown stimuli. (b) Relative threshold obtained by subtracting the threshold of the White stimulus from Pink and from Brown for each observer.

POA in instantaneous model is not sensitive to variable slopes

Comparison of POA between human and model observers, similar to main text results. Here we used for the instantaneous model psychometric curves with slopes that vary across input signal type. The results reported in the main text were obtained using the average curve over the entire experiment; no significant change was found.

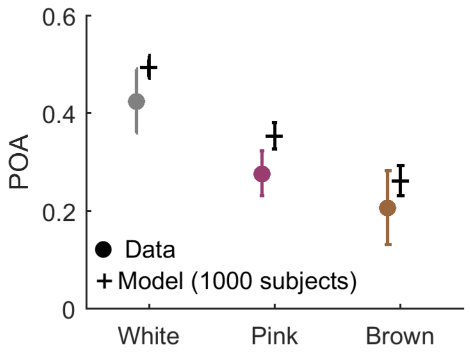

Figure C: POA is not sensitive to differences in psychometric curves among stimuli. In this analysis the curves slope for the instantaneous observer are different for each input mode. Specifically, we used $k_w = 29.3, k_p = 32.8, k_b = 36.5$ for White, Pink and Brown respectively, which are the average slopes over observers in each regime. The results are very similar to those in the main text where $k = 30$ in all cases

Models of Separate Biases

Two partial models were tested separately, with only one of the two biases incorporated in each. One model accounts for the positive recency bias only, and the other for the long-term adaptation only. These two effects are combined in the model that was presented in the main text in section and its performance is presented in the figures below.

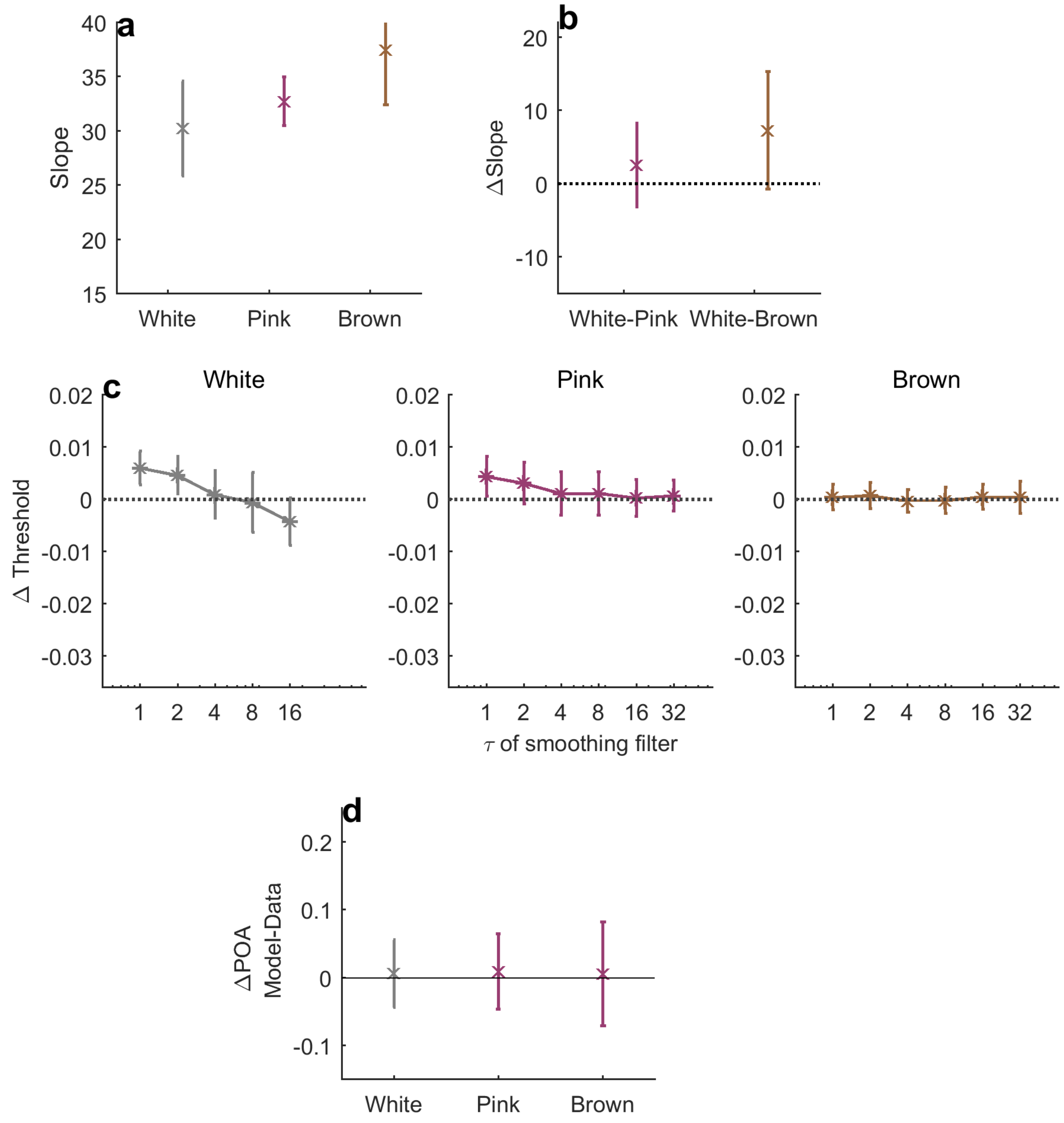

Figure D: OnlyRecency (a) and (b) demonstrate the effect on the slope of the psychometric curve. (c) The hysteresis with respect to direction of input trends is missing the negative phase that dominates in the long τ values in the data. (d) POA averaged in each stimulus regime was used to calibrate the model with the correct strength of positive recency. The difference between the POA in this partial model to the actual data is therefore zero within accruacy of the measurements.

Model of Adaptation Bias Only

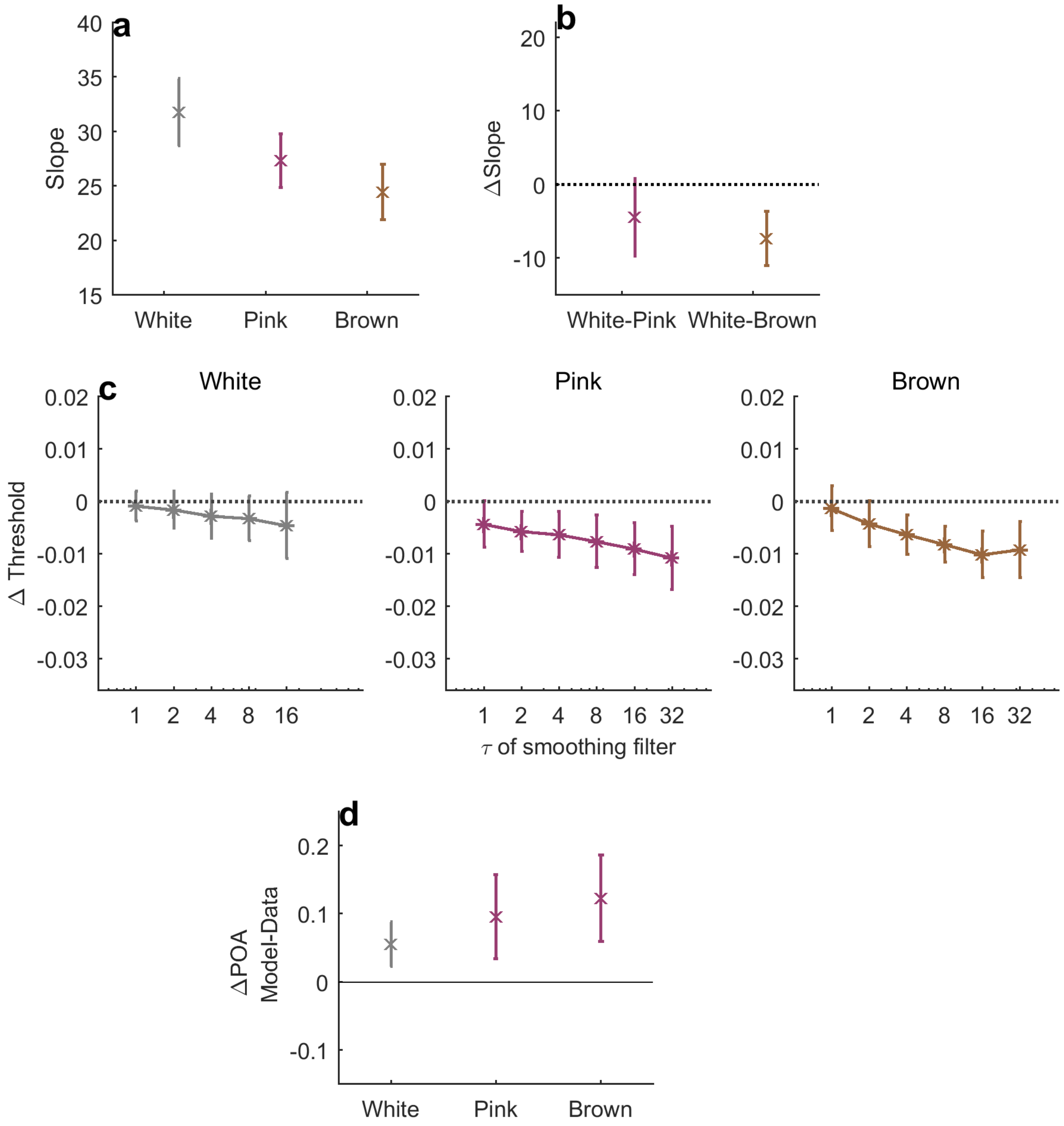

Figure E: OnlyAdaptation (a) and (b) The effect on the slope of the psychometric curve is opposite to the measured effect, decreasing instead of increasing. (c) Threshold hysteresis with respect to input trend is negative in all τ values, unlike the measured results. (d) The difference between this partial model and the data POA is increasing, reflecting higher alternation rates than as correlation time increases. This is in qualitative disagreement with the data.

Other models tested

Bias modulations by response only This model is similar to the one presented in the main text, only with the adaptation bias regulated by the history of responses rather than of inputs. The output y is filtered with a time constant τ to give $\bar{y}_i$, which replaced $\bar{x}_i$ in the adaptation variable A. The performance the two models is almost identical, however we find this configuration of feedbacks to be less plausible physiologically.

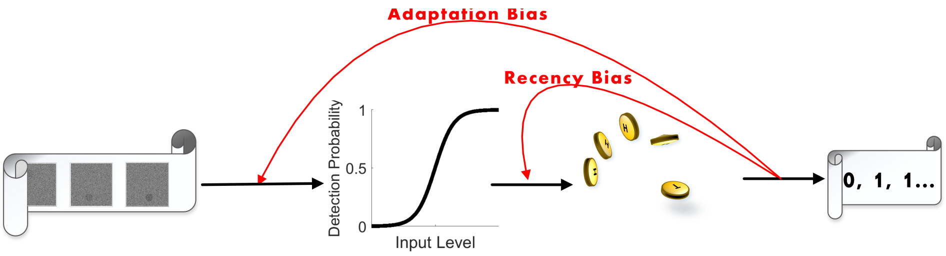

Figure F: Bias modulations by response only.

Bias modulations of output only

In this model the two biases are introduced in the post-sensory stage of processing. Sensory processing is instantaneous and independent on history. It is modeled by constant sigmoidal relations between the momentary input level and the probability of the coin flip. The decision itself, on the other hand, encapsulates all history dependencies. The performance of this model was inferior to the one presented in the main text in reproducing the experimental results, and moreover parameter values needed to be tuned and the results were less robust.

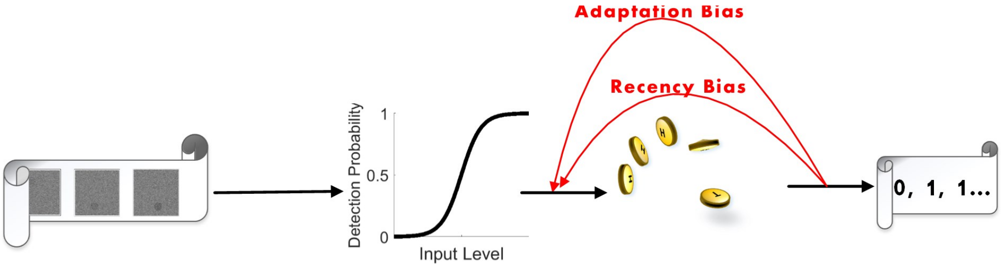

Figure G: Bias modulations of output only

Spatial Effects

Here we tested the influence of spatial proximity on the response. Different distances in pixels were used to define spot as "close"/”far" to the previous one. A reduction of threshold was found for spatially close-by spots, (example in Figure Ha(a)), but only when spot location was very close, less than a half the spot size (distance<=30 pixels between centers) [Druker, 2010]. The effect reduced rapidly with distance (Figure H(b)), diminishing completely when no overlap existed between consecutive stimuli (distance>60 pixels). Trials where the distance between consecutive spots was <=30 pixels constitute only 15% of all trials (Figure H(c)). Therefore, this small fraction alone cannot account for the global effect of adaptation.

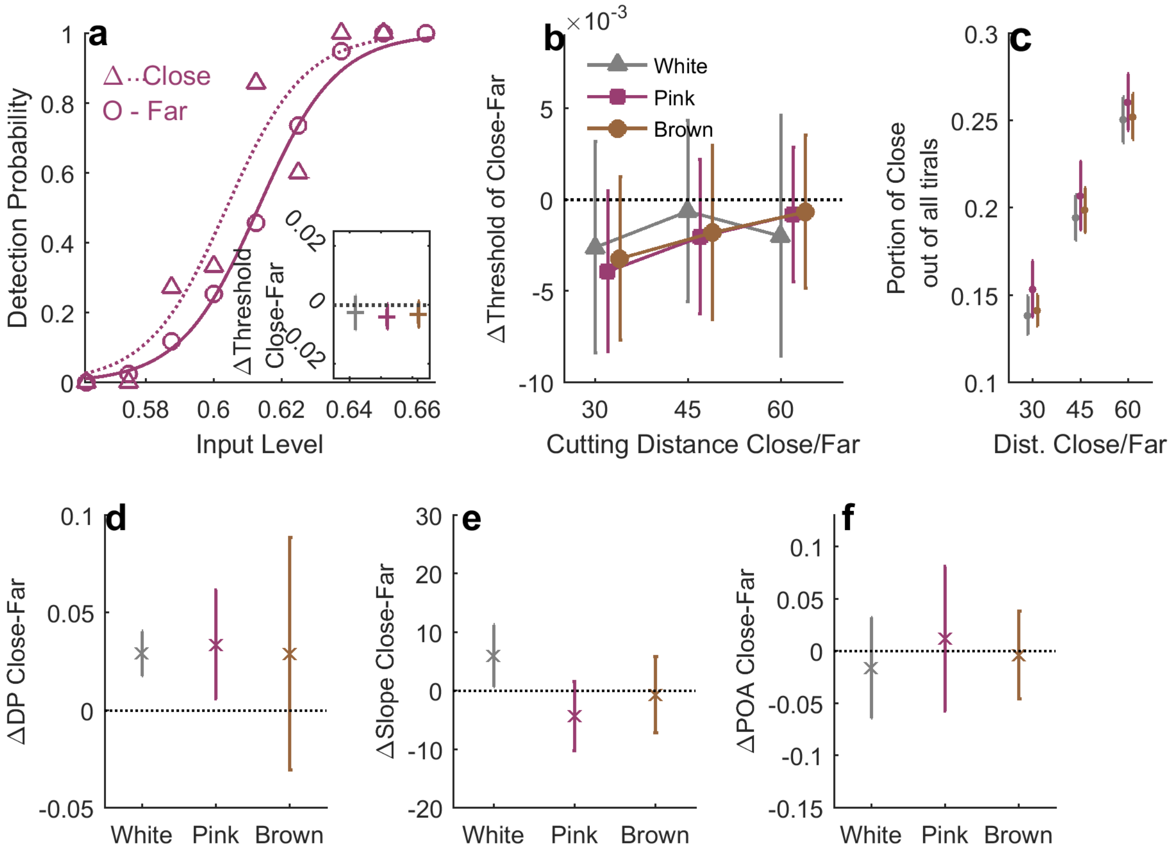

Figure H: Spatial Effects (a) Example of threshold reduction by consecutive overlapping spots, with less than 30 pixels distance between centers (triangles), relative to the rest of the trials (circles). Insert: for all observers the thresholds psychometric curves of "far" stimuli were subtracted from those of the "close" ones. The differences were overall small and negative. (b) This spatial effect was tested with various threshold distances. The differences were detected only in the very close distances, when the consecutive spots were partially overlapping (distance<=30 pixels).(c) The fraction of "close" distances out of all trials, as a function of defined pixel overlap. (d) The detection probability of very short distances between consecutive trials is slightly higher than these of the long distances. The individual differences between the DP are plotted for of short of distances <30 pixels, i.e. less than half overlap between the consecutive trials. (e) and (f) no consistent effects on slope and probability of alternation were found.

References

[Druker, 2010] Druker (2010). Spatial probability aids visual stimulus discrimination. *Front. Hum. Neurosci.*, 4(August):1–10.
